# Modeling clinical and non-clinical determinants of intrapartum stillbirths in singletons in six public hospitals in the Greater Accra Region of Ghana: a case–control study

**DOI:** 10.1038/s41598-022-27088-9

**Published:** 2023-01-18

**Authors:** Linda Vanotoo, Duah Dwomoh, Amos Laar, Agnes Millicent Kotoh, Richard Adanu

**Affiliations:** 1 Office of the Country Director of USAIDs Health System Strengthening Accelerator Programme, Accra, Ghana; 2 Department of Biostatistics, School of Public Health, University of Ghana, Accra, Ghana; 3 Department of Family and Reproductive Health, School of Public Health, University of Ghana, Accra, Ghana

**Keywords:** Biomarkers, Health care, Medical research

## Abstract

The Greater Accra Region (GAR) of Ghana records 2000 stillbirths annually and 40% of them occur intrapartum. An understanding of the contributing factors will facilitate the development of preventive strategies to reduce the huge numbers of intrapartum stillbirths. This study identified determinants of intrapartum stillbirths in GAR. A retrospective 1:2 unmatched case–control study was conducted in six public hospitals in the Greater Accra Region of Ghana. A multivariable binary logistic regression model was used to quantify the effect of exposures on intrapartum stillbirth. The area under the receiver operating characteristics curve and the Brier scores were used to screen potential risk factors and assess the predictive performance of the regression models. The following maternal factors increased the odds of intrapartum stillbirths: pregnancy-induced hypertension (PIH) [adjusted Odds Ratio; aOR = 3.72, 95% CI:1.71–8.10, *p* < 0.001]; antepartum haemorrhage (APH) [aOR = 3.28, 95% CI: 1.33–8.10, *p* < 0.05] and premature rupture of membranes (PROM) [aOR = 3.36, 95% CI: 1.20–9.40, *p* < 0.05]. Improved management of PIH, APH, PROM, and preterm delivery will reduce intrapartum stillbirth. Hospitals should improve on the quality of monitoring women during labor. Auditing of intrapartum stillbirths should be mandatory for all hospitals and Ghana Health Service should include fetal autopsy in stillbirth auditing to identify other causes of fetal deaths. Interventions to reduce intrapartum stillbirth must combine maternal, fetal and service delivery factors to make them effective.

## Introduction

Most pregnancies progress normally from conception to delivery. However, others do not go through smoothly because of antepartum or intrapartum maternal, fetal, and health sector factors that place the mother, the fetus, or both at a higher risk for developing complications than pregnancies without those factors^1^. Such pregnancies lead to the death of the babies, mothers, or both by the end of pregnancy and delivery^2,3^. A stillbirth is the death of a baby after 28 weeks of pregnancy and can occur either before or during labour and delivery^4^. Intrapartum stillbirth is defined as fetal deaths that occur during labour or delivery where the baby does not show any signs of skin disintegration and is clinically described as fresh stillbirths^5,6^. Recent global estimates have revealed that intrapartum stillbirths as a single birth outcome exceed the contribution of malaria to child deaths^7^. Globally in 2019, an estimated 2·0 million babies were stillborn at 28 weeks or more of gestation, over 90% occurred in low income countries and more than 40% of stillbirths occur intrapartum^4^. Stillbirth rates in 2019 varied widely across regions, from 22·8 stillbirths per 1000 total births in west and central Africa^4^.

Of the 650,000 deliveries that were recorded annually in public health facilities in Ghana, 1.7% ended as stillbirths with 40% occurring intrapartum^8^.

Stillbirth has not been widely studied in low and middle-income countries (LMICs) especially in settings where obstetric care is readily available ^9^ and it has not been considered as part of major global maternal and child health strategies, although it is a reflection of the quality of a country’s healthcare system for pregnant women^8,10^. Cross-sectional studies conducted in low- and middle-income countries have identified some obstetric complications during the intrapartum period, such as preeclampsia, fetal mal-presentation, prolonged labor, preterm delivery, or cesarean section as some of the factors associated with intrapartum stillbirth^11–13^. Administration of two or more doses of oxytocics to augment labour before reaching the facilities can lead to intrapartum stillbirths^14^. Maternal factors that impact birth outcomes include young or advanced maternal age, use of drugs and smoking during pregnancy, untreated hypertension during pregnancy, maternal infections such as malaria, syphilis and HIV, maternal conditions especially hypertension, and diabetes, other complications during labour, traveling for more than 30 min to reach a health facility from home, health professionals taking more than 10 min to attend to the pregnant woman after reaching the facility, presence of any complication during labour and delivery, warranting treatment and poor access to caesarean section when needed contribute to intrapartum stillbirth^15,16^. Fetal factors that contribute to intrapartum stillbirth include post-maturity, fetal growth restriction (when an unborn baby is unable to achieve its growth potential and therefore smaller than it should be) and congenital abnormalities^16^. Other studies have linked intrapartum stillbirth with poverty in Ghana^17^.

A clear understanding of the factors that contribute to these intrapartum stillbirths will facilitate the development of interventions to inform policy, program, and practice to reduce preventable intrapartum stillbirths. Developed or high-income countries have all birth and deaths registered and these countries rely on the information from health facilities to guide the development of interventions aimed at reducing intrapartum and neonatal deaths^9^. However, in most LMIC, there is inadequate vital registration system on perinatal and neonatal deaths and these countries rely on public health surveillance such as demographic and health surveys, maternal health surveys and multiple indicator cluster surveys^9^ but most of these surveys do not contain enough information to understand the factors that contribute to intrapartum stillbirth. This study aimed to use facility-based data to assess how maternal, fetal, and service delivery factors contribute to intrapartum stillbirths in order to identify potentially preventable deaths.

## Methods

### Study design

The study employed a 1:2 unmatched case–control study design where two controls were matched to one case. A census of all intrapartum stillbirths recorded between 1st January and 31st December 2016 in the six selected health facilities was carried out. A total number of 362 intrapartum stillbirths were reported in the six hospitals’ documentation between the study periods. However, out of the 362 cases, records were available for only 125 cases. This resulted in the selection of 250 controls using systematic random sampling. These 250 controls were randomly sampled from a total number of 35,250 live births recorded within the study period. The number of controls selected per hospital was based on the number of cases selected from that hospital. The background characteristics of the cases and controls were obtained from the hospital records. Some of the reasons for the inability to trace the folders include the relocation of the records department at the hospital and folders were given to clients to take home. All the 125 cases were therefore studied. Although the census of all cases was conducted, an ex-post power analysis was conducted to determine whether the study design has been powered enough to detect a significant effect of the predictors of interest. The estimated power was 82% based on 125 cases and 250 controls assuming a type I error of 5% and the approximate odds ratio of 2%.

The informed consent of the participants was waived by the Ghana Health Service Ethical Review Committee because the patients’ folders were de-identified.

All procedures of the study were carried out in accordance with relevant institutional and national guidelines and regulations.

This study followed the standard guidelines for reporting observational studies using the strengthening the reporting of observational studies in epidemiology (STROBE).

### Study area

The study was conducted in the Greater Accra Region of Ghana within six public hospitals that provide antenatal, delivery, and post-natal services for pregnant women, both as primary and referred clients. The six hospitals were: Greater Accra Regional Hospital, Ridge; Tema General Hospital; La General Hospital; Ga South Municipal Hospital; Ledzekuku Krowu Municipal Assembly (LEKMA) Hospital and Achimota Hospital. These hospitals were purposively selected because the six hospitals provide a similar range of out-patient and in-patient services; they have the expertise to conduct delivery and carry out interventions including cesarean sections in women with complications during labour. The hospitals have the highest number of Doctors, Specialists Obstetricians/Gynaecologists, Midwives, Nurses, Pharmacists, Anesthesiologists, and professional Health Service Administrators working within GHS in GAR. All the hospitals are secondary level district facilities with referral networks to tertiary facilities. All of them are in the capital city of Ghana and have relatively mid to high volume delivery. The only exception is the Greater Accra Regional Hospital which is a regional level facility with a high volume delivery and also receives referrals from all the other hospitals.

They also have well-established functional governance/management structures and are major contributors to the huge numbers of stillbirths in GAR.

### Study population

The study population was all women with singleton pregnancies at 28 weeks or more gestation, who delivered in the six selected public health facilities in 2016. The delivery and post-natal case-folders of these women formed the basis for the selection of cases and controls.

### Inclusion criteria

The cases and controls were selected based on a set of criteria developed before data collection began. The criteria are: women with singleton pregnancies of 28 or more weeks gestation; women who delivered in any of the 6 selected public health facilities in 2016; women with live births (controls) delivered at 28 or more weeks gestation in 2016; women who delivered intrapartum stillborn babies (babies with no signs of maceration or skin disintegration (cases) at 28 or more weeks gestation in 2016.

### Exclusion criteria

The following criteria were set to exclude potential participants. Women who qualified to be part of the study based on our inclusion criteria but folders could not be traced at the time of the study.

### Outcome measure

The primary outcome measure of interest was intrapartum stillbirth defined as the delivery of any fetus after 22 weeks of gestation, or with a birth weight more than 500 g, who had detectable fetal heart sounds upon admission, but died during the intrapartum period and thus had an Apgar score of 0 at 1 and 5 min, without signs of maceration^18^.

### Exposure variables

The following variables were studied based on the availability of information in the patient's folder and evidence from previous studies that have identified them as potential risk factors^9,19^: age of the mother, education level, place of residence, marital status, employment, parity, weight at registration, hemoglobin at first registration, previous history of stillbirth, history of neonatal deaths, antenatal care attendance, intermittent preventive treatment, fever, malaria, diabetes, hypertension, pregnancy-induced hypertension (systolic blood pressure > 140 mmHg and diastolic blood pressure > 90 mmHg), eclampsia, premature rupture membrane (is a rupture of the membranes or the amniotic sac before labor begins), antepartum hemorrhage and duration of labour in hours.

### Data collection and management

Well-trained midwives were used to retrieve patients records from the hospital folders. Data were collected using a standardized questionnaire designed to reflect the information on patients’ folders and entered in excel. Seven different research assistance were used to verify the information from the patients' folders that were entered in excel. The cleaned version of the data was exported to Stata for statistical analysis.

### Data analysis

In determining the relationship between intrapartum stillbirth and each of the categorical covariates, bivariate analyses were conducted using Fisher’s exact test and Pearson Chi-square test of independence. Fisher’s exact test was used when more than 5% of the expected frequencies in a two by two contingency table was less than five. In comparing quantitative continuous and discrete covariates between mothers who experienced stillbirth (cases) and those who did not (controls), the Welch t-test and the Wilcoxon Rank Sum test were used where appropriate. In the case of the non-normally distributed covariate, the Wilcoxon rank-sum test was used to compare the median of the covariate under investigation between the cases and the control groups. The Welch t-test was used to compare the mean of quantitative covariates between the cases and controls for normally distributed covariates. Although the study adopted a predictive modeling approach to identify potential risk factors, we emphasized that prediction could be considered as a superset of hypothesis testing and estimation. Details on why we prefer a model-based approach to address our research objective over techniques that only yield *p*-values have been documented elsewhere^20^.

Twenty-one multiple imputations by chained equations (MICE) were used to impute missing values. The number of imputations was arrived at by comparing Markov Chain Monte Carlo error on the coefficients. The rule of thumb is that the number of multiple imputations should be increased until the Markov Chain error is 10% or less with a *p*-value less than 0.01. The proportion of missing values was approximately 7%. The ability of fetal, maternal, and service delivery characteristics to predict stillbirths were evaluated individually using area under the receiver operating characteristics curve (AUROC) and the brier score (BS). Since the sample size was small relative to the number of cases (intrapartum stillbirth), only predictors with higher AUROC and smaller BS were used in the multivariable analysis. The AUROC and BS measure the discrimination and calibration performance of each predictor variable. The F-test measures the overall significance of the model. Logistic regression models with robust standard error were fitted to the imputed data sets to investigate the odds of fetal, maternal, and service delivery risk factors that increase the likelihood of a pregnant woman having an intrapartum stillbirth. The odds ratio estimates from the logistic regression model, their respective standard errors, and predictive performance indices were combined using Rubin’s rule^21^. Some variables were dropped in the multivariable analysis because of high covariate imbalance (low variability) between cases and control. These variables included admission during pregnancy, area of residence, history of previous stillbirth, history of neonatal death, evidence of abnormality, fever, syphilis, and malaria status at the time of delivery. For instance, almost all the patients’ folders reviewed showed about 98.0% of mothers (both cases and control) lived in the urban area indicating little variation in the area of residence and hence the decision to drop those variables from the multivariable model to increase the probability of model convergence.

### Ethical approval and consent to participate

Ethical approval was obtained from the Ghana Health Service Ethical Review Committee through the Health Research and Development Division (GHS, RDD). The requirements were duly met as stipulated on the website at http://www.ghanahealthservice.org/publications. The ethical clearance certificate was GHSERC23/06/17.

## Results

### Maternal and fetal characteristics

The number of deliveries recorded in all the six selected hospitals in the Greater Accra Region between 1st January 2016 and 31st December 2016 was 36,168. Out of this 35,250 (97.5%) were live births whilst 918 (2.5%) were stillborn. Also, 362 (39.4%) out of the stillbirths occurred intrapartum. Based on available data, a total of 125 cases (intrapartum stillbirths) and 250 controls (live births) were selected for the study. The mean ages of the women were 28.8 years for the cases and 28.9 years for the controls.

### Association between maternal factors and intrapartum stillbirth

Table 1 shows the bivariate analysis of maternal factors and intrapartum stillbirth. The Pearson Chi-square test of independence showed that pregnancy-induced hypertension; premature rupture of membranes and antepartum hemorrhage were associated with intrapartum stillbirth. Among mothers who delivered intrapartum stillbirth, 27.0% had experienced pregnancy-induced hypertension compared to 8.1% in the control (*p* < 0.001). Among the cases, 8.0% had experienced premature rupture of membranes compared to 4.0% in the controls (*p* < 0.001). With regards to antepartum hemorrhage, 24.8% of the cases had previously experienced intrapartum stillbirths compared to 2.5% among the controls (*p* < 0.001).

**Table 1:** Bivariate analysis of maternal factors associated with intrapartum stillbirth in the Greater Accra Region in 2016.

Maternal characteristics	Control		Cases		*p*-value
	No. of mothers (n = 250)	n (%)	No. of mothers (n = 125)	n (%)	
**Age in years** (mean + SD)	244	28.88 ± 5.54	117	28.85 ± 6.05	0.957
**Age categories**					
< 35 years	244	205 (84.02)	117	92 (78.63)	0.842
> = 35 years		39 (15.98)		25 (21.37)	
**Educational level**					
None	177	13 (7.34)	60	8 (13.33)	0.198
Primary		53 (29.94)		11 (18.33)	
Secondary		90 (50.85)		35 (58.33)	
Tertiary		21 (11.86)		6 (10.00)	
**Marital status**					
Married	237	206 (86.92)	114	95 (83.33)	0.273
Single		31 (13.08)		19 (16.67)	
**Area of residence**					
Urban	244	242 (99.18)	117	113 (96.58)	0.071
Rural		2 (0.82)		4 (3.42)	
**Employment**					
Unemployed	220	14 (6.36)	101	11 (10.89)	0.160
Formal employment		26 (11.82)		12 (11.88)	
Informal employment		180 (81.82)		78 (77.23)	
**Parity**: median (LQ, UQ)	236	1.00 (1.00, 2.50)	120	2.00 (1.00,3.00)	0.299
**Parity categorize**					
No child	236	55 (23.31)	120	29 (24.17)	0.856
1–4		168 (71.19)		86 (71.67)	
4 +		13 (5.51)		5 (WHO)	
**Weight at registration** (mean ± SD)	6	75.01 ± 18.57	25	64.37 ± 14.62	0.231
**Hemoglobin at 1st registration** (mean ± SD)	6	10.57 ± 1.36	29	10.71 ± 1.61	0.829
Hemoglobin: median (LQ, UQ)	2	6.88 (6.65,7.10)	15	6.05 (4.95,6.60)	0.052
**Age between last child and current Pregnancy**: median (LQ, UQ)	154	2.00 (0.00,4.00)	78	2.00 (0.00,5.00)	0.485
**History previous stillbirth**					
Yes	245	3 (1.22)	115	4 (3.48)	0.467
No		242 (98.78)		111 (96.52)	
**History of neonatal deaths**					
Yes	239	5 (2.09)	111	4 (3.60)	0.470
No		234 (97.91)		107 (96.40)	
**ANC attendant**					
Yes	231	225 (97.40)	109	103 (94.50)	0.526
No		6 (2.60)		6 (5.50)	
**Number of ANC Visits** (mean ± SD)	7	5.57 ± 2.51	30	4.55 ± 2.09	0.367
**Number of ANC visits**					
0–3 visits	7	2 (28.57)	38	13 (34.21)	0.197
> 3 visits		5 (71.43)		25 (65.79)	
**IPT doses** (mean ± SD)	15	2.20 ± 1.08	34	2.24 ± 0.82	0.245
**Fever**					
Yes	246	6 (2.44)	67	3 (4.48)	0.410
No		240 (97.56)		64 (95.52)	
**Malaria**					
Yes	248	3 (1.21)	63	3 (4.76)	0.100
No		245 (98.79)		60 (95.24)	
**Diabetes**					
Yes	248	1 (0.40)	98	0	1.000
No		247 (99.60)		98 (39.00)	
**Hypertension**					
Yes	248	2 (0.81)	100	4 (4.00)	0.100
No		246 (99.19)		96 (96.00)	
**PIH**					
Yes	248	20 (8.06)	100	27 (27.00)	< 0.001
No		228 (91.94)		73 (73.00)	
**Eclampsia**					
Yes	247	3 (1.21)	98	2 (2.04)	0.625
No		244 (98.79)		96 (97.96)	
**Admission in pregnancy**					
Yes	247	8 (3.24)	67	2 (2.99)	1.000
No		239 (96.76)		65 (97.01)	
**PRM**					
Yes	242	10 (4.13)	125	10 (8.00)	< 0.001
No		232 (95.87)		81 (64.80)	
**Antepartum hemorrhage**					
Yes	236	6 (2.54)	125	31 (24.80)	< 0.001
No		230 (97.46)		79 (63.20)	
**Labor duration in hours**: median (LQ, UQ)	129	8.40 (5.00,13.56)	59	7.30(3.35,15.00)	0.300

*PIH* Pregnancy Induced Hypertension, *PRM* Premature Rapture of Membrane, *SD* Standard Deviation, *UQ* Upper quartile, *LQ* Lower quartile, (%) represent column percentage; n: Total number of cases and controls that were used in the estimation of the test statistics.

### Association between fetal factors and intrapartum stillbirth

Table 2 shows the relationship between fetal factors and intrapartum stillbirth. This study investigated seven fetal characteristics and how they could influence the risk of intrapartum stillbirth in the Greater Accra Region. The Welch t-test showed a statistically significant difference in the mean gestational age of fetuses between mothers that had previously experienced intrapartum stillbirth and those that have not had such experience. The mean gestational age of the fetus in weeks for mothers that have not experienced stillbirth was approximately 38.8 weeks compared to those that have experienced stillbirth, which was 35.6 weeks (*p* < 0.001). The sensitivity analysis based on the Chi-square test of independence when gestational age in weeks was re-categorized into preterm and term births also showed a significant relationship with intrapartum stillbirth (*p* < 0.001). Birth weight was statistically different between the cases and controls. The mean birth weight of stillbirths (cases) was generally lower compared to the birth weight of babies born alive (controls) (2.7 kg versus 3.1 kg, *p* < 0.001). There was an association between birth weight and intrapartum stillbirth (*p* < 0.001). Among mothers who had an intrapartum stillbirth, 37.6% of them delivered babies with low birth weight while 15.4% in the control delivered babies with low birth weight. Evidence of abnormality, characteristics of the liquor and placenta had a statistically significant relationship with intrapartum stillbirth (*p* < 0.05).

**Table 2:** Fetal factors associated with intrapartum stillbirth in six public hospitals in 2016.

Fetal characteristics	Control		Cases		*P*-value
	Number of mothers (n = 250)	n (%)	Number of mothers (n = 125)	n (%)	
**Sex of child**					
Male	244	135 (55.33)	110	36 (57.27)	0.120
Female		109 (44.67)		47 (42.73)	
**Gestational age** (mean ± SD)	203	38.80 ± 2.69	103	36.54 ± 3.95	< 0.001
**Gestational age categorized**					
Preterm (Below 37 week)	203	52 (25.62)	103	53(51.46)	< 0.001
Term (37 weeks and above)		151 (74.38)		50 (48.54)	
**Birth weight **(mean ± SD)	240	3.08 ± 0.65	109	2.70 ± 0.92	< 0.001
**Birth weight (Collins et al.)**					
Low (< 2.5 kg)	240	37 (15.42)	109	41 (37.61)	< 0.001
Normal ($\ge$ 2.5 kg)		203 (84.58)		68 (62.39)	
**Position of the baby**					
Otherwise	241	12 (4.98)	115	21 (18.26)	< 0.001
Cephalic		229 (95.02)		94 (81.74)	
**Evidence of abnormality**					
Yes	245	3 (1.22)	120	14 (11.67)	< 0.001
No		242 (98.78)		106 (88.33)	
**Characteristics of the placenta**					
Described	247	176 (71.26)	105	50 (47.62)	< 0.001
Not Described		71 (28.74)		55 (52.38)	
**Characteristics of liquor**					
Clear	224	206 (91.96)	32	16 (50.00)	< 0.001
Meconium Stained		18 (8.04)		16 (50.00)	

*SD* Standard Deviation. (%) represent column percentage, *n* Total number of cases and controls that were actually used in the estimation of the test statistic.

### Association between service delivery factors and intrapartum stillbirth

Table 3 shows the relationship between service delivery factors and intrapartum stillbirth. This study investigated three service delivery factors that can influence intrapartum stillbirth. These factors were the mode of delivery, the category of a health professional who conducted the delivery, and the time of delivery. The Pearson Chi-square test analysis showed that there is a relationship between the mode of delivery, a health professional who conducted the delivery, and intrapartum stillbirth (*p* < 0.05). Among the cases, 51.2% of babies were delivered via cesarean section compared to 34.1% delivered by cesarean section among the controls (*p* < 0.001). Approximately 55.2% of cases were delivered by medical doctors compared to 39.1% of deliveries among the controls (*p* < 0.001). The time of delivery was not found to be statistically significant. Mode of delivery and the health professional who conducted the delivery could be associated with intrapartum stillbirth but caution must be taken in the interpretation of these results since this relationship was only observed during the bivariate analysis.

**Table 3:** Bivariate analysis of service delivery factors associated with intrapartum stillbirth in six public hospitals in 2016.

Service delivery factors	Control		Cases		*P*-value
	Number of mothers (n = 250)	n (%)	Number of mothers (n = 125)	n (%)	
**Mode of deliver**					
CS	247	84 (34.01)	121	62 (51.24)	< 0.001
SVD		163 (65.99)		59 (48.76)	
**Person conducted the delivery**					
Medical Officer	220	86 (39.09)	116	64 (55.17)	< 0.001
Midwife		134 (60.91)		52 (44.83)	
**Time of delivery**					
Day shift	230	152 (66.09)	117	74 (63.25)	0.280
Night shift		78 (33.91)		43 (36.75)	

*CS* Caesarian Section, *SVD* spontaneous vaginal delivery; (%) represent column percentage, *n* Total number of cases and controls that were used in the estimation of the test statistic.

### Analysis of the predictors of intrapartum stillbirth

The results from the AUROC and Brier score showed that only six out of the 14 maternal factors investigated met the criteria for inclusion into the multivariable model; they had relatively higher AUROC and smaller Brier Score as shown in Table 4. Concerning fetal factors, after the rigorous variable screening to determine fetal factors to be included in the multivariable analysis using AUROC and Brier Score, gestational age was the only fetal factor that met the criteria of inclusion into the multivariable logistic regression model (AUROC = 67.0%, Brier score = 0.1979) (Table 4). Regarding health service delivery factors, the discriminating and calibration indices of these service delivery factors (mode of delivery, a health professional who conducted the delivery) did not meet the criteria for inclusion into the multivariable logistic regression model. They were therefore excluded in the multivariable model to avoid a spurious relationship with intrapartum stillbirth and to improve the parsimony of the model.

**Table 4:** Screening variables to be included in the multivariate analysis using the area under receiver operating characteristics curve and the Brier score.

Variables	AUROC [95% CI]	BRIER SCORE
**Fetal factors**		
Sex of child	0.50 [0.45, 0.56]	0.2220
Gestational age of child weeks	0.67*** [0.61, 0.74]	0.1979
Birthweight of child in kg	0.64 [0.57, 0.70]	0.2080
Intra uterine position	0.57 [0.53, 0.60]	0.2087
**Maternal factors**		
ANC	0.54 [0.50–0.58]	0.2199
Age of mother	0.51 [0.44, 0.57]	0.2222
Marital status	0.53 [ 0.49–0.58]	0.1887
Mothers educational status	0.67 [0.61, 0.73]	0.2211
Parity of mother	0.53 [0.47, 0.60]	0.2216
Antepartum Haemorrhage	0.64^§^ [0.60, 0.69]	0.1940
Haemoglobin at registration	0.81^§^ [0.76, 0.85]	0.2132
Premature rupture of membrane	0.64^§^ [0.60, 0.68]	0.1942
Pregnancy induced hypertension	0.71^§^[ 0.64–0.77]	0.1886
Weight at Registration in kg	0.71^§^[0.66, 0.76]	0.2076
IPT doses	0.81^§^ [0.76, 0.85]	0.2143
**Service delivery factors**		
Mode of Delivery	0.61 [0.56, 0.66]	0.2177
Time shift delivery	0.51 [0.46, 0.57]	0.2233
Delivered by:	0.58[0.52, 0.64]	0.2166

*AUROC* Area under receiver operating curve. *CI* confidence interval. ^**§**^ indicates variables included in the multivariable analysis, *IPT* Intermittent Preventive Treatment.

### Quantifying the effect of fetal and maternal factors on intrapartum stillbirth

The results from the multivariable logistic regression can be found in Table 5. Controlling for gestational age of the fetus and other maternal characteristics in the logistic regression model, among mothers who experienced pregnancy-induced hypertension, the odds of experiencing intrapartum stillbirth was approximately 4 times the odds of experiencing intrapartum stillbirth among women without a history of pregnancy-induced hypertension (aOR = 3.7, 95%:1.71–8.1, *p* < 0.01; Table 5). Among women who experienced antepartum hemorrhage, the odds of intrapartum stillbirth was approximately 3 times the odds of intrapartum stillbirth among women without antepartum hemorrhage (aOR = 3.28, 95% CI:1.33–8.1, *p* < 0.05; Table 5). Among women who had premature rupture of membranes, the odds of experiencing intrapartum stillbirth was approximately 3.4 times the odds of experiencing intrapartum stillbirth in women who did not experience premature rupture of membranes (aOR = 3.36, 95% CI: 1.2–9.4, *p* < 0.05; Table 5). Controlling for key maternal characteristics, the results showed that a unit increase in gestational age in weeks is associated with approximately 17% reduction in intrapartum stillbirths (aOR = 0.83, 95% CI: 0.73–0.96, *p* < 0.01).

**Table 5:** Quantifying the effect of fetal and maternal factors on intrapartum stillbirth.

	**A binary logistic regression model with robust standard error**	
	Model 1	Model 2
	aOR [95% CI]	aOR [95% CI]
Gestational age of the child	0.86** [0.79–0.94]	0.83** [0.73–0.96]
**Antepartum hemorrhage**		
No	ref	ref
Yes	2.85** [1.43–5.71]	3.28* [1.33–8.10]
**Premature-rupture membrane**		
No	ref	ref
Yes	3.28** [1.59–6.75]	3.36* [1.20–9.40]
**Pregnancy induced hypertension**		
No	ref	ref
Yes	3.59*** [1.80–7.17]	3.72** [1.71–8.10]
Hemoglobin at first registration	#	1.13 [0.72–1.79]
Weight at first Registration	#	1.00 [0.97–1.02]
IPT doses		1.35 [0.66–2.75]
**Model performance index**		
AUROC [95% CI]	0.73*** [0.66, 0.80]	0.85***[0.81- 0.90]
Brier score	**0.1566**	**0.1323**

*ref* reference category. *OR* Odds Ratio. *P*-value notation: ***: *p* < 0.001. **: *p* < 0.01. *p < 0.05. *CS* caesarian section. *SVD* spontaneous vagina delivery; #: Not included in model 1. *IPTp* Intermittent Preventive Treatment for pregnant women, *AUROC* Area under the Receiver Operating Characteristics Curve.

### Combinations of key risk factors in predicting intrapartum stillbirth

**Figure 1:**
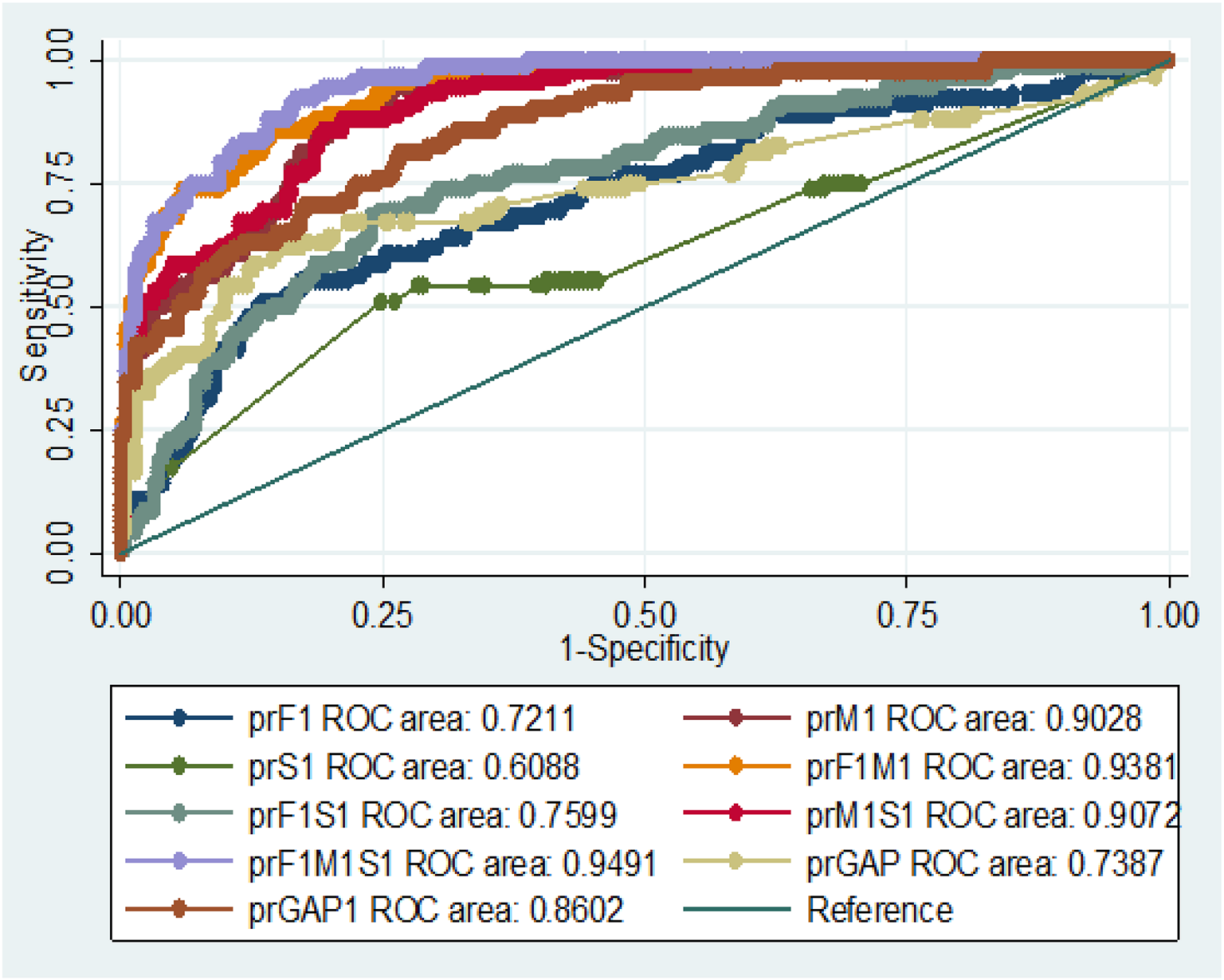
Assessing discrimination ability of nine different models on intrapartum stillbirth: *ROC* Receiver Operating Characteristics Curve; *prF1* Only Fetal factors; *prM1* Only Maternal factors; *prS1* Only Service factors; *prF1M1* Fetal + Maternal factors; *prM1S1* Maternal + Service factors; *prF1M1S1* Fetal + Maternal + Service factors; *prGAP* Gestational age + antepartum Hemorrhage + pregnancy induced hypertension + pre-rapture membrane; *prGAP1* Gestational age + antepartum Hemorrhage + pregnancy induced hypertension + pre-rapture membrane + Hemoglobin at registration + Weight at registration + Intermittent Preventive Treatment for pregnant doses.

To identify the combination of predictors (fetal, maternal, and service delivery factors) that could discriminate between mothers who are likely to have a stillbirth and those who will not, calibration estimates from the Brier Score and the AUROC were used. AUROC is the probability that if you were to take a random pair of women, one who has experienced stillbirth and one without such experience, the woman who has had stillbirth will have a higher predicted risk than the other. The AUROC thus gives the probability that the model correctly ranks such pair of women. From the nine models compared (Fig. 1 and Supplemental Table S1), the model with fetal, maternal, and service delivery factors had the highest AUROC and the smallest Brier Score indicating that it is the best model to probably predict stillbirth (AUROC = 95.0%, Brier score = 0.0907).

## Discussions

This study set out to examine the maternal, fetal, and health service factors that contribute to intrapartum stillbirths in singleton pregnancies. This study investigated 14 different maternal factors that could increase the delivery of intrapartum stillbirth including maternal age, education, place of residence, and occupation.

### Maternal sociodemographicfactors

The study did not identify any significant association between maternal sociodemographic characteristics such as age, level of education, place of residence, occupation, and intrapartum stillbirth. These results are contrary to findings from other studies that have investigated the effect of maternal socio-demographic characteristics on stillbirth and reported that low maternal education, age above 35 years, residing in rural areas; maternal illiteracy, and unemployment were significant risk factors for delivery of intrapartum stillbirth^22,23^. Advanced maternal age (> 35 years) is associated with fetal anoxia resulting from poor placental blood flow and this may explain the association between older age and intrapartum fetal death^24^. Advanced maternal age has also been associated with the possibility of chronic diseases in the mother such as hypertension which affects uteroplacental blood flow^25^. The lack of sufficient statistical evidence of an association between sociodemographic characteristics and intrapartum stillbirths in this study may be due to the geographical location of GAR. The Greater Accra Region is the Capital of Ghana and the effect of development and urbanization has influenced the living conditions of the subjects thus removing the influence of sociodemographic characteristics on intrapartum stillbirths. For instance, GAR is a metropolitan and has numerous health facilities making access to care during pregnancy relatively easier. The impact of the geographic location could be seen in this study as aoproximately 95% of cases and 98% of controls attended ANC, and all of them delivered in a hospital.

### Antepartum hemorrhage

The anterior position of the placenta during pregnancy negatively affects pregnancy outcomes more than when the placenta is placed in the posterior position. In this study 24.80% of the cases had APH compared to 2.54% in the controls; however, the records in the women’s folders did not state whether they were due to placenta praevia or abruptio placentae. APH was found to be a statistically significant factor associated with intrapartum stillbirth implying that women who experienced APH were at a higher risk of having intrapartum fetal death compared to women who did not have APH. This finding is consistent with findings from other researches which have reported APH as a significant risk factor for intrapartum stillbirth^22^. Placentation in the anterior position can go on to cover the cervix and this can lead to bleeding and poor perinatal outcomes such as intrauterine fetal growth restriction, preterm delivery, and premature rupture of membranes^26^. It is one of the complications in pregnancy that is an important cause of perinatal mortality and maternal morbidity^27^. The two major causes of APH are placenta praevia and abruptio placentae and together they contribute about 50% of all cases of APH. In the other 50% of cases, the cause is difficult to identify even after investigations^27^. This is consistent with findings from other researches that 51.6% of pregnant women with placenta praevia develop APH^28^. However, findings from other researchers found that about 9.8% of women with intrapartum fetal deaths had placental abruption which was lower than what others have reported^29^.

### Pregnancy-induced hypertension, pre-eclampsia, and eclampsia

Pregnancy-induced hypertension and eclampsia are associated with poor pregnancy outcomes for both the mother and the newborn. In the newborn, they contribute significantly to stillbirth as a significant proportion of perinatal deaths. For instance, in a study on women diagnosed with pre-eclampsia, the researchers reported as much as 84.5% of the perinatal deaths were stillbirths^30^. Pregnancy-induced hypertension was found in 13.5% of the total study population and this was higher than 7.5% reported in a prospective cohort study on pregnant women in two antenatal clinics in Accra^31^. In this current study, among the cases, 27.0% experienced pregnancy-induced hypertension compared to 8.1% in the control group and this was statistically significant as a predictor of intrapartum stillbirth. This finding of significance is consistent with what was reported in other studies in Accra^32^. A study in Zimbabwe found that women who developed PIH during pregnancy were 4.3 times more likely to have stillbirth as a pregnancy outcome compared to women who did not develop PIH^33^.

### Gestational age

Preterm is defined according to the ICD-10 version 26, as a baby born at or after 28 completed weeks but before 37 completed weeks (196 completed days but less than 259 completed days) of gestation^34^. Increased gestational age was found to be associated with lower odds of intrapartum stillbirth. These findings are consistent with reports from Dassah et al.^32^ whose work was also done in Accra, that preterm delivery, hypertensive disorders in pregnancy, breech delivery, and vacuum extraction were significant risk factors for stillbirths delivery (aRR, 5.15; 95% CI, 4.18–6.35). The findings are also consistent with reports from India, which globally has the highest absolute number of stillbirths that preterm delivery is one of the significant risk factors for stillbirth^35^.

### Strengths of the study

This study is one of the few studies that have examined both clinical and non-clinical determinants of intrapartum stillbirths in GAR. The unique feature of this study is that it examined maternal, fetal, and service delivery factors that contributed to intrapartum stillbirths in the GAR. Another strength is that the study used a census of available records on intrapartum stillbirths to identify the possible contributing factors of intrapartum stillbirths in GAR.

### Limitations of the study

The major limitation of the study was the challenge of retrieving stored data for both the cases and controls. This is a limitation of retrospective studies and an example of poor data management practices that occur in health facilities especially in developing countries. To address this shortcoming, the study employed multiple imputations by chained equations (MICE) to input missing data before the multivariable analysis was conducted; thus increasing the validity of the findings. Another limitation is that autopsy for stillbirths is not a routine practice in Ghana therefore, this study did not have access to fetal predictors that can only be obtained through autopsy reports and lack of autopsy may lead to misclassification of the outcome (that is, misclassifying stillbirth as intrapartum or vice versa). The study utilized records on the physical findings of the baby at birth to determine whether a baby had congenital abnormalities or not in the absence of autopsy reports. The same assessment was used for live babies, thus there was no discrimination between determining abnormalities in stillbirths and live births. Midwives and doctors can examine babies at birth and determine and document whether babies are born with congenital abnormalities or not. Therefore, the reports on abnormalities reported in both the cases and controls can be said to be professionally plausible. A fetal autopsy is not regularly performed in developing countries. Agbata et al.^36^ reported that although fetal autopsy examination remains the best tool for understanding and classifying the causes of stillbirth it has not been used extensively in developing countries. Since the study examined several contributing factors including congenital abnormality and used robust statistics to identify significant factors that contribute to intrapartum fetal deaths, the absence of autopsy reports would not invalidate the findings of the study.

## Conclusions

The study identified maternal, fetal, and health service factors that contribute to intrapartum stillbirths using a more rigorous statistical model. Pregnancy-induced hypertension (PIH), antepartum hemorrhage (APH), premature rupture of membranes (PROM) were key maternal factors identified as contributors to intrapartum stillbirths in singletons. The gestational age of the fetus at birth was the key fetal factor that contributed significantly to intrapartum stillbirths. Health service delivery factors such as mode of delivery and health professional who conducted the delivery were not found to be statistically significant during the multivariate analysis. The best model to predict intrapartum stillbirth was the model which combined maternal, fetal, and service delivery factors compared to models which had individual contributory factors or models with combinations of two contributory factors. The GHS should review ANC education provided to pregnant women and emphasize the identification and prompt response to complications in pregnancy. All doctors, midwives, and nurses who care for pregnant women should be trained in the proper management of pregnancy-induced hypertension (PIH); antepartum hemorrhage (APH), and premature rupture of membranes (PROM) to improve quality of care and birth outcome. The GHS should develop SOPs on proper management of preterm labour and delivery, train health care providers to use the standards, and regularly monitor adherence to improve quality of care and birth outcomes. As a matter of urgency, the Ministry of Health should review the curriculum of health training institutions to give priority attention to the management of PIH, APH, PROM, and prematurity to ensure that staff enter the service with the requisite knowledge to improve the quality of care of pregnant women.

## Supplementary Information

Supplementary Information.

## Data Availability

The datasets generated and/or analyzed during the current study are not publicly available due to legal and ethical concerns but are available from the corresponding author on reasonable request.

## Abbreviations

APH: Antepartum hemorrhage
ANC: Antenatal care
GHS: Ghana health service
PIH: Pregnancy-induced hypertension
PROM: Premature rupture of membranes
ROC: Receiver operating characteristics curve
SOP: Standard operating procedure
