# Supplementary material for: Modeling clinical and non-clinical determinants of intrapartum stillbirths in singletons in six public hospitals in the Greater Accra Region of Ghana: a case–control study
Source: Sci Rep. 2023 Jan 18;13:1013. doi: 10.1038/s41598-022-27088-9 (PMC9849331; doi:10.1038/s41598-022-27088-9)
Supplement: Supplementary file 1 — Supplementary Information. [file 41598_2022_27088_MOESM1_ESM.docx]

**Modeling clinical and non-clinical determinants of intrapartum stillbirths in singletons in six public hospitals in the Greater Accra Region of Ghana: a case-control study**

Table S1: Calibration and discrimination ability of nine different models on intrapartum stillbirth

| Factors studied | AUROC | 95% CI | Brier Score |
| --- | --- | --- | --- |
| Model 1: Unique selection 1: Gestational age + antepartum Hemorrhage + pregnancy induced hypertension + pre-rapture membrane | 0.74 | 0.67-0.81 | 0.1566 |
| Model 2: Unique selection 2: Gestational age + antepartum Hemorrhage + pregnancy induced hypertension + premature-rupture of membrane + Hemoglobin at registration +Weight at registration + Intermittent Preventive Treatment doses | 0.86 | 0.82-0.90 | 0.1323 |
| Model 3: Only Fetal factors | 0.72 | 0.66-0.79 | 0.1887 |
| Model 4: Only Maternal factors | 0.90 | 0.87-0.94 | 0.1239 |
| Model 5: Only Service delivery factors | 0.61 | 0.54-0.68 | 0.2134 |
| Model 6: Fetal + Maternal factors | 0.94 | 0.91-0.96 | 0.0976 |
| Model 7: Fetal + Service delivery factors | 0.76 | 0.70-0.82 | 0.1853 |
| Model 8: Maternal + Service delivery factors | 0.91 | 0.88-0.94 | 0.1177 |
| Model 9: Fetal + Maternal + Service factors | 0.95 | 0.93-0.97 | 0.0907 |

AUROC: Area Under the receiver operating characteristics curve: Ranges from 0.5 (50%) to 1.0 (100%), the higher the better; Brier score measures the losses incurred in using the model to predict stillbirth, it ranges from 0 to 1, the smaller the better.
